# Regression of Moral Reasoning during Medical Education: Combined Design Study to Evaluate the Effect of Clinical Study Years

**DOI:** 10.1371/journal.pone.0017406

**Published:** 2011-03-30

**Authors:** Darko Hren, Matko Marušić, Ana Marušić

**Affiliations:** 1 University of Split Faculty of Philosophy, Split, Croatia; 2 Department of Research in Biomedicine and Health, University of Split School of Medicine, Split, Croatia; Yale University School of Medicine, United States of America

## Abstract

**Background:**

Moral reasoning is important for developing medical professionalism but current evidence for the relationship between education and moral reasoning does not clearly apply to medical students. We used a combined study design to test the effect of clinical teaching on moral reasoning.

**Methods:**

We used the Defining Issues Test-2 as a measure of moral judgment, with 3 general moral schemas: Personal Interest, Maintaining Norms, and Postconventional Schema. The test was applied to 3 consecutive cohorts of second year students in 2002 (n = 207), 2003 (n = 192), and 2004 (n = 139), and to 707 students of all 6 study years in 2004 cross-sectional study. We also tested 298 age-matched controls without university education.

**Results:**

In the cross-sectional study, there was significant main effect of the study year for Postconventional (F(5,679) = 3.67, P = 0.003) and Personal Interest scores (F(5,679) = 3.38, P = 0.005). There was no effect of the study year for Maintaining Norms scores. 3^rd^ year medical students scored higher on Postconventional schema score than all other study years (p<0.001). There were no statistically significant differences among 3 cohorts of 2^nd^ year medical students, demonstrating the absence of cohort or point-of-measurement effects. Longitudinal study of 3 cohorts demonstrated that students regressed from Postconventional to Maintaining Norms schema-based reasoning after entering the clinical part of the curriculum.

**Interpretation:**

Our study demonstrated direct causative relationship between the regression in moral reasoning development and clinical teaching during medical curriculum. The reasons may include hierarchical organization of clinical practice, specific nature of moral dilemmas faced by medical students, and hidden medical curriculum.

## Introduction

Newly graduated physicians start their Hippocratic Oath with words: “I swear to fulfil, to the best of my ability and **judgment**, this covenant …”. In their daily work they will encounter a plethora of ethical problems [1], so the ‘judgment’ part of their oath will certainly include a moral judgment, i.e. moral reasoning. According to the cognitive-developmental approach based on Kolberg's ideas [2], the development of moral reasoning occurs through change in the proportions of three cognitive schemas used while reasoning about a moral dilemma [2]. ‘Personal Interest’ is the least developed schema which is characterized by thinking about personal gains or losses of each participant of the moral dilemma or their significant others. The next and more advanced, in terms of fairness and justice, is the ‘Maintaining Norms’ schema, characterized by realization that one needs to get along with people other than friends and kin, and therefore needs rules and norms to stabilize behaviours and expectation among people who are not familiar intimates and may have different interests. Finally, the most developed moral reasoning uses ‘Postconventional’ schema, characterized by the primacy of moral criteria, appeal to shareable ideals and full reciprocity. According to the theory, individuals irreversibly progress from using mostly ‘Personal Interest’ towards using mostly ‘Postconventional’ schema when thinking about a moral dilemma [2], [3]. The critical period of transition to the postconventional moral reasoning is late adolescence and young adulthood [3], [4]. In this period, educational experience can play an essential role and the majority of studies confirmed the positive association between moral reasoning and higher education [4], [5].

The evidence for the relationship between higher education and moral reasoning does not, however, clearly apply to medical students. Previous studies showed that the advancement in moral reasoning does not occur during medical school [6]–[9] or that it may even decrease [10]–[12]. Although without empirical evidence, some authors argued that the indicated plateau or regression in moral reasoning during medical studies may be due to students' experiences in the clinical part of their study [7], [13]. To test the causative relationship between clinical part of the medical curriculum and the development of moral reasoning, we used the combined study design derived from Schaie's description of a general model for study of developmental problems [14].

## Methods

### Participants and study design

**Figure 1:**
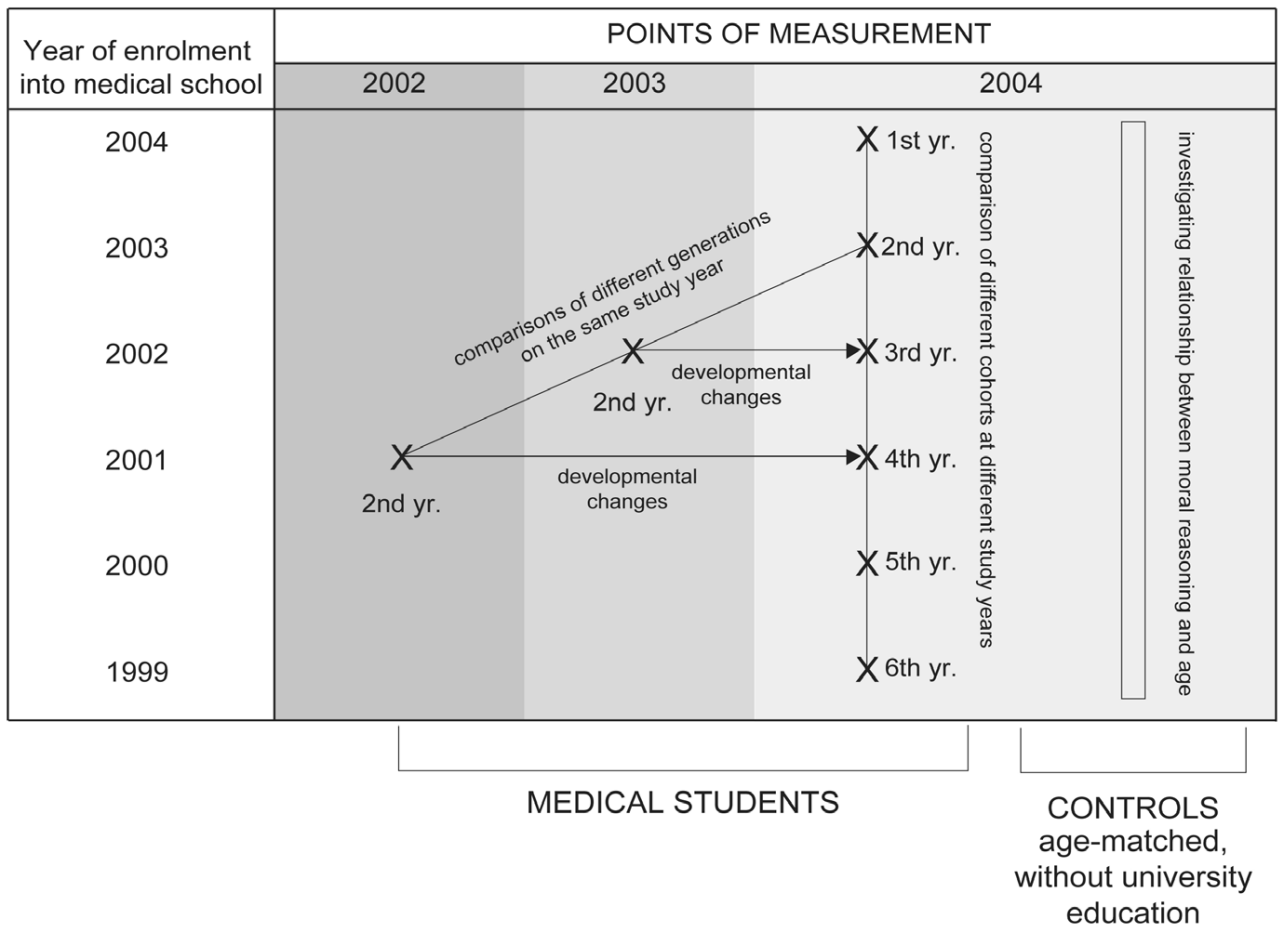
Study design.

When used to investigate changes over time, cross sectional studies confound age and cohort differences, whereas longitudinal studies confound age and time-of-measurement effects [14]. To address this problem we complemented them by a time-lag study design, which compares samples of individuals of the same age at different time points (Figure 1).

In the fall of 2002, we tested 207 2^nd^ year medical students (62% women, median age = 20, interquartile range = 0, min-max = 19–25). Testing was anonymous, and confidentiality was assured by unique identification code chosen by a student, to enable re-testing.

At the same time in 2003, we tested 192 students from the 2^nd^ year cohort (63% women, median age = 20, interquartile range = 0, min-max = 19–27) using the same procedure, and in the fall of 2004 we tested 707 medical students from all 6 study years (68% women, median age = 21, interquartile range = 2, min-max = 18–27). The 3^rd^ and 4^th^ year students were asked in 2004 to use a code because these were students who were already tested in 2 previous studies. Based on the matching codes we paired scores for 75 students first tested in 2002 and 61 students first tested in 2003.

Using this approach we had scores from 3 generations of 2^nd^ year medical students, which allowed us to test whether there was a stable pattern of development. We also had cross-sectional data from medical students of all 6 years, which allowed us to investigate possible differences between students on different study years. Finally, we had repeated measurements on two cohorts of students, which provided information about changes over time: 75 matched repeated measurements at 2^nd^ and then 4^th^ study year and 61 matched repeated measurements at 2^nd^ and 3^rd^ study year. We chose the 3^rd^ and 4^th^ study years for repeated measurements because this is the change from preclinical (up to the 3^rd^ year) to clinical part of the curriculum (4^th^ to 6^th^ year) at the University of Zagreb School of Medicine [15].

Additionally, we included a control group of participants to investigate whether maturation in age had an effect by itself on moral reasoning in this age group. The control group included students from Zagreb Public Educational Centre, who attended classes for vocational re-training (n = 298, 37% female, median age = 21, interquartile range = 4, min-max = 18–27). The enrolment criterion for this group was that a participant never attended any university programme. To ensure the same age span (18–27 years) for the control and medical student groups, 4 medical students over 27 were excluded from the study. Additional 24 tests (17 medical students and 7 controls) were excluded from the analyses due to incomplete or invalid data.

The participation in the study was voluntary and anonymous. The respondents were informed about the general purpose of the survey and were assured that all measures were taken to ensure the anonymity of the process. The participants were not asked for a written consent and filling out the questionnaire was considered as the consent for the study. The Ethics Committee of the Zagreb University School of Medicine approved the study, including the consent procedure.

### Instrument

We used the Defining Issues Test-2 (DIT-2), a paper-and-pencil self-administrative test of moral judgment derived from Kohlberg's theory [16]. It presents five hypothetical dilemmas and asks the respondent to rate and rank 12 issues in terms of their importance for each dilemma. The scores represent the degree to which a respondent uses 3 general moral schemas in reasoning about a moral dilemma: arguments that appeal to personal interests (Personal Interest), maintaining social laws and norms (Maintaining Norms), or moral ideals and/or theoretical frameworks for resolving complex moral issues (Postconventional Schema). A confirmatory factor analysis of a mega-sample of over 44000 subjects demonstrated that DIT items cluster around these three general moral schemas [3]. The instrument also provides information about participant's developmental profile by indicating which schema predominates in a respondent's moral reasoning and whether he or she is consolidated in that schema or in transition to a higher reasoning schema. The information about predominant schema in first measurement was used in the longitudinal part of the study to assess whether initial schema preference influenced challenge patterns in repeated measurements.

The validity of the DIT has been thoroughly investigated in terms of 7 criteria [16]: 1) differentiation of various age/education groups, where 30% to 50% of the variance of DIT scores is attributable to education level; 2) longitudinal gains, which show effect sizes of 0.80 in freshmen to senior college students, making gains in DIT scores one of the most dramatic effects of college; 3) significant relation to cognitive capacity measures of Moral Comprehension, recall and reconstruction of Postconventional moral arguments, Kohlberg's interview measure, and to a lesser degree to other cognitive developmental measures; 4) sensitivity to moral education interventions; 5) linkage to many ‘prosocial’ behaviours and desired professional decision making; 6) linkage to political attitudes and political choices, with a correlation in the 0.40–0.65 range; and 7) adequate reliability, with Cronbach α and test-retest reliability in the 0.70–0.80 range. Further, the information in a DIT score predicts the 7 validity criteria above and beyond that accounted for by verbal ability or political attitude. The DIT is equally valid for males and females.

DIT-2 is an updated version of the original DIT, with updated stories, shorter test, clearer instructions, retaining of more subjects through reliability checks [16]. With the permission of the Center for the Study of Ethical Development, University of Minnesota, USA, we translated the DIT-2 into Croatian using a back-translation method for all but names of the protagonists and small parts of the stories 2, 3 and 5, which were adjusted to the Croatian social environment without changing the important content of the stories [17]. The data from the Croatian version of the test were copied to original DIT-2 forms, and scored by the Center for the Study of Ethical Development, University of Minnesota, USA.

### Statistical analysis

Participant's age was described using median, interquartile and total range of scores and DIT-2 scores were described using mean and 95% confidence interval for mean.

We used Pearson's r coefficient of correlation to test the association between age and DIT-2 scores, and point-biserial coefficient to test the association between sex and DIT-2 scores. Because we found an association between sex and DIT-2 scores, we included sex as a covariate in all subsequent analyses based on General Linear Model (GLM). One-way ANCOVA was used to test the differences in DIT-2 scores between 3 generations of medical students on 2^nd^ year of study in the time-lag part of our study. The same procedure was used in the cross-sectional part of the study to test the differences between medical students on different study years. We also used a polynomial contrasts analysis to test whether scores follow any developmental trend, such as linear, quadratic, or cubic. Finally, in the longitudinal part of the study, we used a mixed within-between subjects ANCOVA with repeated measurements as within-subjects independent variable, and two between-subjects independent variables: time between the two measurements (one or two years) and schema preference in the first measurement. The assumptions for ANCOVA, normality of distributions, homogeneity of regression lines and homogeneity of variances, were met for all analyses.

All analyses were performed using SPSS 17 for Windows (SPSS Inc., Chicago, IL). The level of statistical significance was set at p<0.05.

## Results

To distinguish potential effects of age from effects of studying we first tested the relationship between age and DIT-2 scores within medical students and controls. There were no significant correlations between age and DIT-2 scores (Postconventional schema, Pearson's r = 0.07 (P = 0.069) for students, r = 0.02 (P = 0.783) for controls; Maintaining Norms, r = 0.01 (P = 0.868) and r = 0.04 (P = 0.498); and Personal Interest, r = 0.01 (P = 0.884) and r = 0.06 (P = 0.331), respectively), demonstrating that maturation in age did not play a significant role in development of moral reasoning in the investigated age group and that potential differences in moral reasoning scores of medical students should be attributed to their educational experience.

We also tested the relationship between gender and DIT-2 scores, as literature showed that women tend to score higher than men [18]. We found low but statistically significant correlation in the expected direction (Postconventional schema, Point biserial correlation 0.20 (p<0.001) for students, 0.12 (p<0.001) for controls; Maintaining Norms, 0.12 (P = p<0.001) and 0.04 (P = 0.524); and Personal Interest 0. 41 (p<0.001) and 0.12 (p<0.001), respectively). Gender was therefore included as a covariate in all subsequent analyses. The assumptions for performing the ANCOVA, i.e. normality of distributions, homogeneity of regression lines and homogeneity of variance, were met for all analyses.

### Time-lag design

We compared 3 groups of second year medical students for their scores on DIT-2 in order to investigate potential cohort or point-of-measurement effects. There were no statistically significant differences among the three groups of second year medical students for any of the three DIT-2 scores (Table 1).

**Table 1:** Average values (95% confidence intervals) of DIT-2 scores* for 3 cohorts of 2^nd^ year medical students.

DIT-2 schema	Cohort			F(2,534)†	p
	2002 (n = 207)	2003 (n = 192)	2004 (n = 139)		
Postconventional	35.2 (33.6–36.8)	33.7 (31.9–35.5)	32.3 (30.2–34.4)	2.75	0.094
Maintaining Norms	29.3 (27.7–30.9)	30.9 (29.1–32.7)	29.2 (27.4–31.0)	1.17	0.311
Personal Interest	27.2 (25.5–28.9)	26.6 (24.9–28.3)	28.0 (25.9–30.1)	2.26	0.105

*Possible score range 0–100.

†One-way ANCOVA.

### Cross-sectional design

**Figure 2:**
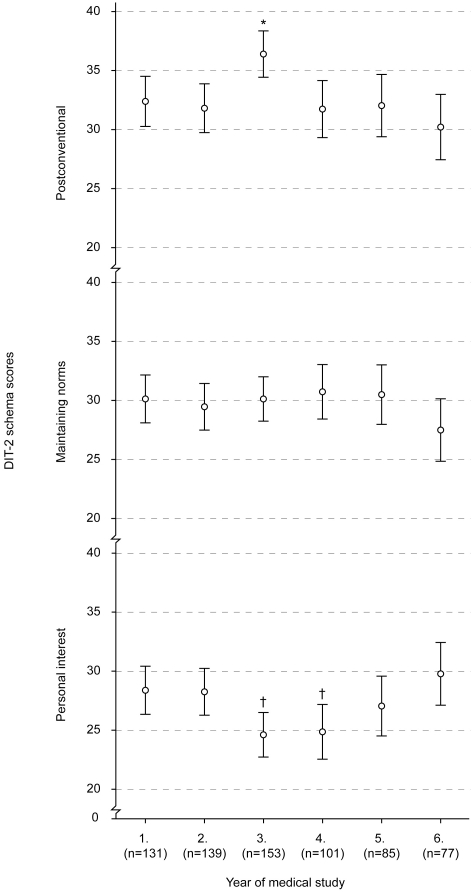
DIT-2 scores (mean±95% confidence interval, CI) of medical students from all six study years. * – p<0.001 vs all other study years; † – p<0.001 vs 1^st^, 2^nd^ and 6^th^ study year.

We found statistically significant main effect of the study year for Postconventional DIT-2 schema scores and Personal Interest scores (Figure 2). There was no effect of the study year for Maintaining Norms scores (Figure 2). Post-hoc analyses showed that 3^rd^ year medical students scored higher on Postconventional schema score than all other study years (p<0.001 for all comparisons), and that 3^rd^ and 4^th^ year students had significantly lower Personal Interest scores than first, second and sixth year students (p<0.001 for the three mentioned comparisons) (Figure 2).

We also performed a polynomial contrast analysis for trend because visual inspection of the scores in Figure 2 suggested that they followed a quadratic trend (increase followed by a decline) for Postconventional scores and reverse quadratic trend (decline followed by an increase) for Personal Interest scores. Both trends were confirmed: Postconventional scores followed a quadratic trend through the 6 study years (contrast estimate = −2.6, P = 0.035), and Personal Interest scores followed a reverse quadratic trend (contrast estimate = 4.1, p<0.001).

### Repeated measurements

Two student generations were tested twice, at different time intervals. Students who were first tested in 2002 (Cohort I) were tested after a 2 year period, and students first tested in 2003 (Cohort II) were tested again after a single year. This allowed us to include time interval as an independent variable in the analysis to evaluate possible differences in score changes due to the time period between measurements. As an additional independent variable, we included DIT-2 schema preference at the first measurement to investigate whether they interacted with score changes.

**Figure 3:**
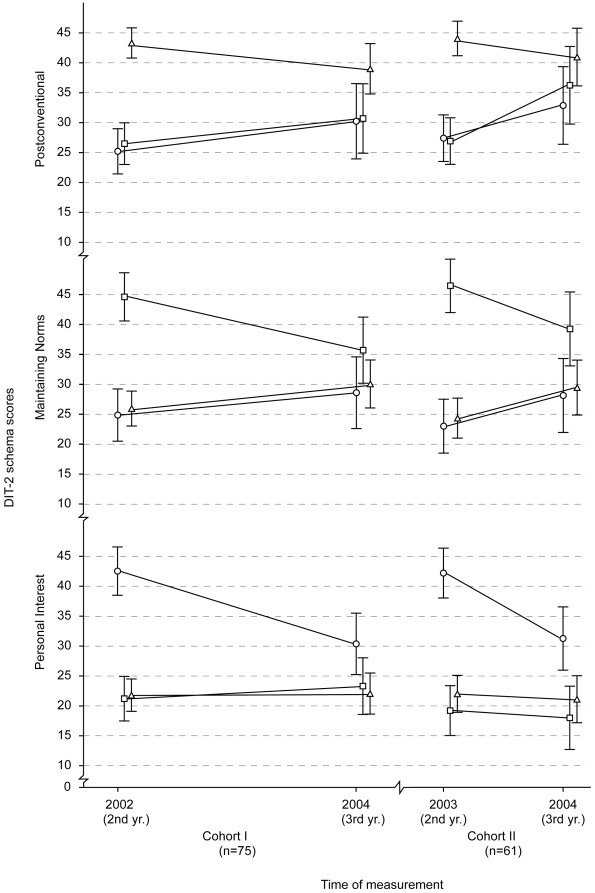
DIT-2 scores (mean±95% CI) in repeated measurements on 2 cohorts of medical students first tested on their 2^nd^ study year and then on 4^th^ year (Cohort I) or 3rd year (Cohort II). Triangles – Postconventional developmental profile, squares – Maintaining Norms developmental profile, and circles – Personal Interest developmental profile at the first measurement point.

There was no main effect of the repeated measurements or the Postconventional schema (F(1,129) = 1.40, P = 0.239) or interaction between time interval and repeated measurements, (F(1,129) = 0.48, P = 0.488) (Table 2). This meant that, when all students from each generation were taken together, there was no significant change in their average Postconventional scores. However, there was a significant interaction between a student's schema preference at the first measurement and score changes in repeated measurement F(1,129) = 8.25, p<0.001, partial η^2^ = 0.11) This demonstrated differences in the direction of changes in Postconventional scores among the 3 groups according to initial schema preference (Figure 3): students who predominantly used Personal Interest or Maintaining norms schema at the first measurement had higher average scores in the second measurement, and the ones who initially used Postconventional schema had lower average scores for that scheme in the second measurement. This interaction also explained the lack of main effect of repeated measurements because different direction of changes between the groups cancelled the overall change. Finally, there was no significant interaction among repeated measurements, initial schema preference, and time interval (F(1,129) = 0.37, P = 0.690), demonstrating that this pattern of change was the same for both generations of students, those tested at the 2^nd^ and then the 3^rd^ year and those tested at the 2^nd^ and 4^t^h year (Figure 3).

**Table 2:** Average values (95% confidence intervals) of DIT-2 scores* in repeated measurements on 2 cohorts of medical students†.

DIT-2 schema	Cohort I (n = 75)		Cohort II (n = 61)	
	2002	2004	2003	2004
Postconventional	34.7 (32.1–37.3)	34.8 (31.7–37.9)	35.2 (32.2–38.2)	37.6 (34.3–40.9)
Maintaining norms	30.7 (28.0–33.4)	31.2 (28.4–34.0)	29.8 (26.3–33.3)	31.7 (28.4–35.0)
Personal interest	26.3 (23.6–29.0)	24.3 (21.6–26.9)	26.6 (23.4–29.8)	23.0 (20.3–25.7)

*Possible score range 0–100.

†The results of mixed within-between subjects ANCOVA are presented in the Results section.

We found the same pattern of scores for Maintaining Norms scores as for Postconventional scores. There was no main effect of repeated measurements for Maintaining Norms scores, (F(1,129) = 0.02, P = 0.904) or the interaction between time interval and repeated measurements (F(1,129) = 0.20, P = 0.656) (Table 2). However, we again found a significant interaction between the initial schema preference and repeated measurements (F(1,129) = 12.58, p<0.001, partial η^2^ = 0.16) and no significant interaction among repeated measurements, initial schema preference, and time interval (F(1,129) = 0.03, P = 0.973).

Finally, there was no main effect of repeated measurements for Personal Interest scores (F(1,129) = 2.62, P = 0.108), or interaction between time interval and repeated measurements (F(1,129) = 0.01, P = 0.944) (Table 2). Once again, we found a statistically significant interaction between initial schema preference and repeated measurements (F(1,129) = 14.87, p<0.001, partial η^2^ = 0.19) (Figure 2) and no significant interaction between repeated measurements, initial schema preference and time interval (F(1,129) = 0.48, P = 0.621).

The pattern of changes in Personal Interest scores was different from that for Postconventional and Maintaining Norms. Personal Interest score of participants who initially predominantly used Postconventional or Maintaining Norms schemas did not change at repeated measurements but decreased for participants who had Personal Interest profile in the first measurement.

## Discussion

Our study demonstrated a reciprocal quadratic change in the development of moral reasoning in medical students, where their scores on Personal Interest schema first decreased in the 3^rd^ year then increased steadily to the 6^th^ year, while Maintaining Norms and Post-conventional Scores first increased modestly in the 3^rd^ and 4^th^ year and then decreased steadily to the 6^th^ year. This indicated that students faced with increasingly clinical learning situations regressed in moral reasoning. We also tested the relationship between age and moral reasoning in control subjects without university education and found no correlations. This meant that any potential differences or changes in DIT scores could be attributed to the education process rather than to students' maturation in age. Next, we investigated potential cohort differences by testing three generation of 2^nd^ year medical students. There were no differences in their scores which indicated a uniform developmental pattern. This also allowed us to interpret cross-sectional data in terms of changes, which otherwise would not be possible due to potential cohort differences. Because of its complex methodological approach and large sample, the findings of our study remove many doubts from results of previous cross-sectional and longitudinal studies performed in the USA [6], [8], Canada [12], and Europe [10], [11]. All these studies indicated no changes or a decrease in moral reasoning during medical studies, which is in contrast to the general conclusions of positive association between higher education and moral reasoning [4], [5]. When this paradoxical phenomenon was first demonstrated, some authors offered a speculation that it might be due to students experiences related to clinical rotations [7], [19]. However there were no empirical data to support these claims, so we designed this study to investigate potential changes in students' moral reasoning over the critical period of transition from basic to clinical part of medical study. Students at the Zagreb University School of Medicine have their first clinical experience in a single course during the 3^rd^ year (*Clinical Propaedeutics*), and all courses are clinical from the 4^th^ year on. We first tested two subsequent generations of students on their second year and then we tested one generation after one year and another after two years. We used students' initial predominant schema of moral reasoning to form 3 subgroups and get an insight into specific change dynamics. The repeated measurements showed equally low levels in the Personal Interest scores for students who initially preferred Postconventional or Maintaining Norms schema. However, Personal Interest scores decreased in repeated measurements for the participants who initially preferred that schema. This was an expected result from the viewpoint of “normal” development [3]. Personal Interest is the lowest schema of moral reasoning [3] and one would expect that its use would decrease in those who initially used it and that it would not increase in those students who have already outgrown it. In the case of uninterrupted development, one would expect that students who initially used Personal Interest schema will show an increase in their Maintaining Norms scores, because for them it would be a progress. In students who initially preferred the Maintaining Norms one would expect a decrease, because they should progress towards Postconventional schema. Finally, students who initially preferred the Postconventional schema should show either no change or decrease in their Maintaining Norms scores because they have outgrown this schema and now should only progress in the Postconventional schema of moral reasoning, leaving the remains of previous schemas behind. The first two expectations were confirmed in our study, indicating progress in students who initially used Personal Interest or Maintaining Norms schemas. However, students who started off with Postconventional schema showed an increase in their Maintaining Norms scores, indicating a regression towards this schema. This also meant that their Postconventional scores should be lower in the second measurement, which we confirmed in our study.

Our finding that the levelling or regression in moral reasoning of medical students occurred as a convergence towards Maintaining Norms schema is important from two standpoints. The first is a practical one and concerns educational and curricular interventions and adjustments in medical studies with an aim to foster moral reasoning. The other is a theoretical one and concerns the fact that, according to Piagetan origins of Kohlbergian and neo-Kohlbergian approaches [2], [3], moral development should not regress. We offer three sets of reasons which we believe might contribute to this convergence towards Maintaining Norms-based moral reasoning in medical students. The first one is the hierarchical system in medicine, in which most medical students start off as young idealists [13] but get disillusioned during their study [20]. The first step in this disillusionment is the amount of facts that they have to take in during the preclinical years. Those who managed to “survive” the demands of preclinical years [21] enter clinical rotations. There, instead of the dignity of the white robe, they are faced with being at the very bottom of a rigid hierarchical system where they have to focus on giving the right answer and getting approval from their teachers, whose values and behaviour may differ from theirs [7]. Branch states that the main internal conflict of medical students is between adhering to their inner moral values and functioning within clinical team, which is mostly based on obeying the hierarchy [19]. The solution can be adhering to norms and rules to make surviving and climbing the hierarchical ladder as painless and easy as possible. The second set of reasons is the specific nature of moral dilemmas faced by medical students. Most interventions aimed at medical students' moral development focus on issues from the medical professional practice. Although necessary, these approaches neglect the fact that medical students cannot yet personally relate to these issues and that they are faced with different ethical dilemmas for which they receive no support. In their analysis of incidents which students reported as critical for their professional development, Christiakis and Feudtner offered the taxonomy of specific ethical dilemmas students encounter in their clinical rotations [22]. These dilemmas were related to students' pursuit of experience, differing degrees of knowledge and ignorance among team members, and dealing with disagreement within the hierarchical authority structure of the medical team. In a great majority of cases, students were left alone with these dilemmas, without an opportunity to discuss them or even to share them and see that they are not the only ones with such concerns. Once again, the solution for students, who have not enough personal or relational resources to solve these dilemmas, is to “go with the stream” and obey norms and rules regardless whether they are explicit or implicit. Finally, the third set of reasons related to the hidden medical curriculum, in which students obtain values, attitudes, beliefs and behaviours typical for medical culture and identity [23], [24] in addition to the knowledge and skills of the official curriculum. Very often hidden curriculum offers opposite values from the formal one, which can lead students to perceive their studies as based on inconsistencies, contradictions and double-bind messages. This in turn can lead to moral relativism and cynicism. Cynicism, as one of the dimensions of Machiavellianism [25], is also associated with lower scores on Postconventional schema [17]. Hafferty and Franks argue that medical students suffer from professional insecurity and fear of failure and that they generalize this perceived incompetence as ethical incompetence as well [23]. In this way the norms and values that are being transferred through the hidden curriculum can be seen as morally acceptable because there is no inner reference.

One threat to internal validity of this study could be regression towards mean [26]. Although we cannot exclude it, we believe that our findings did not suffer from it, or at least not significantly: if there were a significant impact of this artefact, we would have observed higher Personal Interest scores in students who had initially used Maintaining Norms and Postconventional moral reasoning. This was not the case, so we believe that the pattern of changes observed in this study reflects a phenomenon unique for the study of medicine in general and for the transition into the clinic in particular. We also obtained a rather low rate of matched participants in the repeated measurements (36% and 31% for the two cohorts, respectively) although the sample sizes were large enough. The attrition was due to organizational issues and we have no reason to believe that there were any systematic factors to produce a selection bias. The results obtained from this experimental setup also confirmed the findings from our previous studies of cross-sectional and prospective design. Final confirmation of our findings could come from repeated studies in other settings.

The limitation of this study is also the fact that it was performed in a single medical school. However, indications of the trend we observed were obtained in other socio-cultural setting, both European [10], [11] and American [6], [12], and they can serve as a support this study's external validity.

There are at least two important reasons why medical students' regression in moral reasoning is an alarming issue for medical educators and medical professionalism. Firstly, moral reasoning is related to moral behaviour [27], and studies showed that up to 25% patients present physicians with some form of ethical problem or dilemma [1], [28]. Moreover, in a recent study of ethical difficulties of European doctors [29], less than one fifth of over 600 participants reported having access to ethics consultation in individual cases. If they cannot find support in their professional environment, it is even more important for physicians to be equipped with their own, inner resources for dealing with such dilemmas. Secondly, even when their patients do not present them with ethical dilemmas, doctors who score higher on measures of moral reasoning tend to also be more competent in their clinical performance [30]–[32]. Without a pretension to offer a finite solution, we put forward several possibilities of fostering medical students' moral reasoning. The first intervention could be ethics courses as a part of medical curriculum, but only if they are delivered early in the curriculum and involve at least 20 hours of case discussions [8] The second and potentially more effective course of action would be helping medical teachers understand the importance of social learning and developing educational interventions, which would allow them to transfer values and moral reasoning skills to their students along with medical knowledge and skills. Finally, work with critical incidents has been shown to be an effective way of dealing with real life ethical dilemmas of medical students [22], [33]. This approach can help trace and deal with the “real stuff” that students face in their clinical rotations. It can also help students to see each other on a more personal level which, in turn, can help develop more compassionate professional view and therefore more sensitivity towards other people beyond just the rules and norms.
